# Esophagobronchial Fistula Associated With Thoracoabdominal Aortic Aneurysmal Disease and Type IA Endoleak: When Dysphagia Precedes Respiratory Failure

**DOI:** 10.7759/cureus.111842

**Published:** 2026-06-30

**Authors:** Melissa Marques, Inês Antunes, Maria Perry Vidal, Rita Alves, Nuno Deveza

**Affiliations:** 1 Intensive Care Unit, Coimbra Local Health Unit, Coimbra, PRT

**Keywords:** aerodigestive fistula, bronchoscopy, critical care, esophagobronchial fistula, tevar, thoracic endovascular aortic repair, thoracoabdominal aortic aneurysm, upper gastrointestinal endoscopy

## Abstract

Esophagobronchial fistula is a rare but life-threatening complication occurring in the setting of thoracoabdominal aneurysmal disease and previous thoracic endovascular aortic repair (TEVAR). We report the case of a 68-year-old man with chronic kidney disease, rheumatoid arthritis, and previous fenestrated/branched endovascular aortic repair (f/bEVAR) for a thoracoabdominal aortic aneurysm (TAAA) who presented with progressive dysphagia, postprandial vomiting, food intolerance, lumbar pain, and worsening dyspnea. Computed tomography (CT) angiography demonstrated extensive thoracoabdominal aneurysmal disease with a persistent type IA endoleak causing marked esophageal compression and the near-complete obliteration of the left main bronchus. Upper gastrointestinal endoscopy confirmed severe non-traversable extrinsic esophageal compression. Following multidisciplinary discussion, the patient underwent proximal TEVAR to exclude the persistent type IA endoleak. During the postoperative course, he remained ventilator-dependent and developed persistent bronchial secretions, recurrent pulmonary infections, and repeated unsuccessful weaning attempts, prompting further investigation. Repeat computed tomography demonstrated a fistulous communication between the esophagus and the left main bronchus. The diagnosis of esophagobronchial fistula was subsequently confirmed by flexible bronchoscopy and upper gastrointestinal endoscopy. This case highlights the importance of recognizing progressive dysphagia as an early manifestation of aneurysmal compression and maintaining a high index of suspicion for aerodigestive fistulization in patients with thoracoabdominal aneurysmal disease who develop persistent bronchial secretions, recurrent pulmonary infections, and repeated unsuccessful weaning from mechanical ventilation.

## Introduction

Esophagobronchial fistula is a rare but life-threatening condition associated with substantial morbidity and mortality [1]. Aerodigestive fistulas comprise distinct entities, including esophagobronchial, bronchoesophageal, aortoesophageal, and aortobronchial fistulas, each with different anatomical relationships, pathophysiological mechanisms, and clinical implications [2]. In adults, esophagobronchial fistulas most commonly arise from malignancy, infection, trauma, or iatrogenic injury. Its occurrence in the setting of thoracoabdominal aneurysmal disease and previous thoracic endovascular aortic repair (TEVAR) is exceptionally uncommon [3].

Proposed mechanisms include progressive aneurysmal enlargement associated with a persistent type IA endoleak, chronic extrinsic compression, ischemic injury, inflammatory changes, infection, and prosthetic erosion [3]. Clinical manifestations are frequently nonspecific. Progressive dysphagia may precede respiratory deterioration because of esophageal compression, whereas persistent bronchial secretions, recurrent aspiration, pulmonary infections, respiratory failure, or difficult ventilatory weaning should raise suspicion of an underlying aerodigestive fistula [4].

Multimodal imaging combined with endoscopic evaluation is essential for establishing the diagnosis, defining the extent of the fistulous tract, and guiding management [5]. Unlike aortoesophageal or aortobronchial fistulas, the present case involved a direct communication between the esophagus and the left main bronchus, while the thoracoabdominal aneurysmal disease represented the underlying pathological process.

We report a rare case of esophagobronchial fistula occurring in the setting of complex thoracoabdominal aneurysmal disease and a persistent type IA endoleak after previous fenestrated/branched endovascular aortic repair (f/bEVAR), highlighting the diagnostic value of computed tomography (CT), bronchoscopy, and upper gastrointestinal endoscopy.

## Case presentation

A 68-year-old man with chronic kidney disease, rheumatoid arthritis, and a complex history of thoracoabdominal aortic aneurysmal disease presented with a one-month history of progressive dysphagia, postprandial vomiting, food intolerance, lumbar pain, and worsening dyspnea. His vascular history included an open infrarenal aortobiiliac bypass, followed by fenestrated/branched endovascular aortic repair (f/bEVAR) for a thoracoabdominal aortic aneurysm (TAAA).

Computed tomography angiography demonstrated progressive aneurysmal enlargement associated with a persistent type IA endoleak after previous f/bEVAR. The aneurysm caused a marked extrinsic compression of the esophagus and a near-complete obliteration of the left main bronchus, with associated distal atelectatic-consolidative changes (Figure 1).

**Figure 1:**
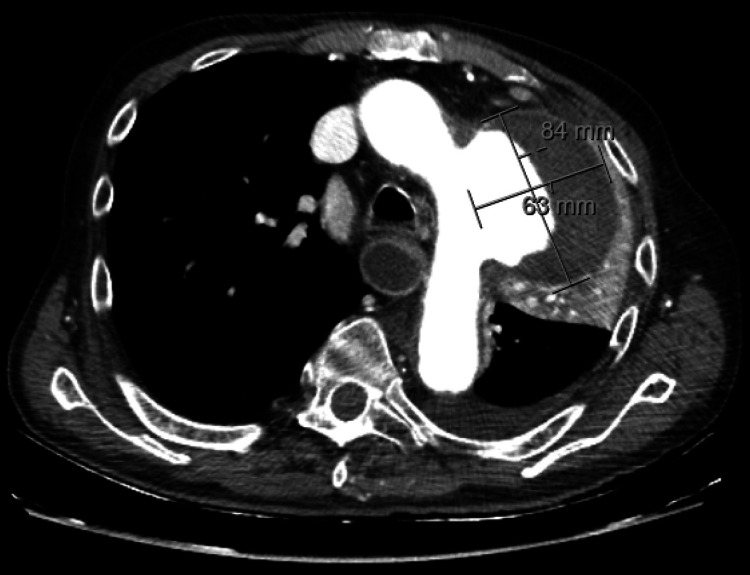
Contrast-enhanced CT demonstrating a giant thoracoabdominal aortic aneurysm (approximately 84 × 63 mm) causing a marked compression of the esophagus and left main bronchus. CT: computed tomography

Upper gastrointestinal endoscopy demonstrated severe non-traversable extrinsic esophageal compression approximately 34 cm from the incisors, consistent with extrinsic compression by the aneurysm (Figure 2).

**Figure 2:**
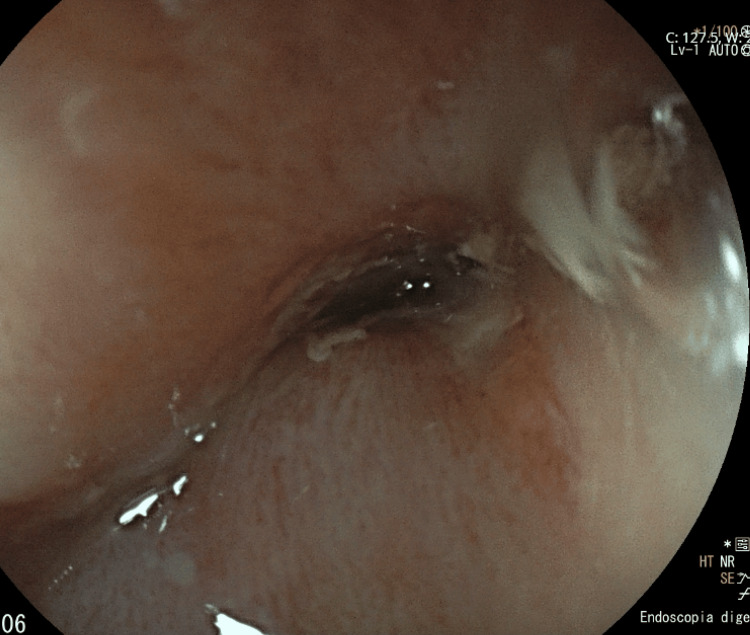
Upper gastrointestinal endoscopy demonstrating severe non-traversable extrinsic esophageal compression.

Following multidisciplinary discussion, the patient underwent proximal thoracic endovascular aortic repair (TEVAR) to exclude the persistent type IA endoleak. Completion angiography confirmed the successful exclusion of the endoleak.

Postoperatively, the patient required intensive care admission because of respiratory failure related to persistent left main bronchial compression, necessitating prolonged invasive mechanical ventilation. During the ICU stay, he developed ventilator-associated pulmonary infection, which was treated empirically with broad-spectrum antibiotics (ceftriaxone, followed by piperacillin/tazobactam and vancomycin), although microbiological cultures remained negative. Fourteen days after TEVAR, despite the successful exclusion of the endoleak, he remained ventilator-dependent and developed persistent bronchial secretions and repeated unsuccessful weaning attempts. Because of persistent respiratory deterioration despite antimicrobial therapy and the successful exclusion of the endoleak, further investigation was undertaken to exclude an aerodigestive fistula.

Repeat contrast-enhanced computed tomography demonstrated a fistulous communication between the esophagus and the posterior membranous wall of the left main bronchus (Figure 3).

**Figure 3:**
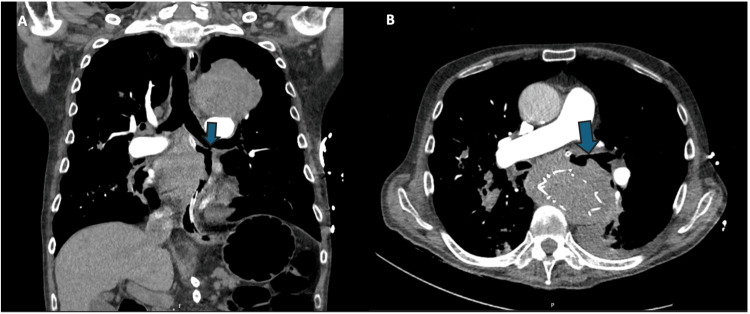
Contrast-enhanced CT demonstrating an esophagobronchial fistula between the esophagus and the posterior membranous wall of the left main bronchus (arrows). CT: computed tomography

Flexible bronchoscopy subsequently confirmed a large fistulous defect in the posterior membranous wall of the left main bronchus, with the direct visualization of the esophageal lumen and the nasogastric tube (Figure 4).

**Figure 4:**
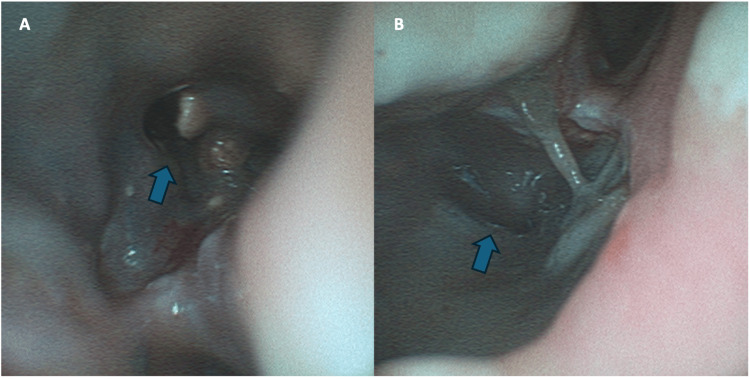
Bronchoscopic view demonstrating the bronchial opening of the esophagobronchial fistula (arrows).

Upper gastrointestinal endoscopy confirmed the esophageal opening of an esophagobronchial fistula measuring approximately 15 mm, with surrounding inflammatory mucosal changes (Figure 5).

**Figure 5:**
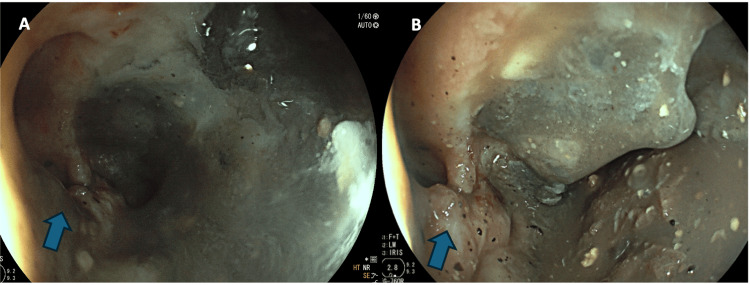
Upper gastrointestinal endoscopy demonstrating the esophageal opening of the esophagobronchial fistula (arrows).

The case was discussed by a multidisciplinary team involving vascular surgery, gastroenterology, interventional pulmonology, and intensive care specialists. Given the complexity of the underlying aortic disease, the extensive fistulous communication involving the left main bronchus, and the patient's overall clinical condition, definitive surgical or endoscopic treatment, including esophageal or airway stenting, was not considered feasible. Despite supportive management, the patient's condition progressively deteriorated, and he died during the same hospital admission.

## Discussion

Esophagobronchial fistula secondary to thoracoabdominal aneurysmal disease is an exceptionally rare complication associated with high morbidity and mortality [3]. Proposed mechanisms include progressive aneurysmal enlargement, chronic extrinsic compression, ischemic injury, inflammatory changes, infection, and erosion related to endovascular prosthetic material, ultimately resulting in communication between the esophagus and adjacent mediastinal structures [3]. Similar mechanisms have been described in reports of aerodigestive fistulas following thoracic endovascular aortic repair (TEVAR), although these complications remain exceptionally uncommon.

In the present case, serial imaging demonstrated progressive aneurysmal enlargement associated with a persistent type IA endoleak, producing a marked compression of both the esophagus and the left main bronchus before proximal TEVAR. Dysphagia and food intolerance preceded respiratory deterioration by several weeks, suggesting that progressive esophageal compression may represent an early warning sign of impending aerodigestive fistulization. Although the fistula was diagnosed after TEVAR, the clinical and imaging findings suggest that it most likely developed in the setting of progressive aneurysmal expansion, persistent type IA endoleak, and chronic mediastinal compression rather than as a direct consequence of the endovascular procedure itself.

Computed tomography angiography is the cornerstone of the initial evaluation, demonstrating aneurysmal anatomy and indirect signs of aerodigestive fistulization, including esophageal compression, bronchial narrowing, mediastinal distortion, and associated pulmonary abnormalities [6]. Bronchoscopy and upper gastrointestinal endoscopy provide complementary information by directly identifying the bronchial and esophageal openings of the fistulous tract, confirming the diagnosis, and defining its anatomical extent [5].

Management requires a multidisciplinary approach involving vascular surgery, thoracic surgery, gastroenterology, interventional pulmonology, and intensive care specialists [7,8]. Therapeutic options include endoscopic esophageal or airway stenting, surgical repair, and additional endovascular interventions; however, treatment is frequently limited by the extent of the fistula, underlying aortic pathology, and patient condition. Consequently, prognosis remains poor, particularly when the central airways are involved [9,10].

This case emphasizes the importance of recognizing progressive dysphagia as a potential early manifestation of aneurysmal compression before respiratory deterioration occurs. In patients with thoracoabdominal aneurysmal disease and previous endovascular repair, persistent bronchial secretions, recurrent pulmonary infections, and repeated failure to wean from mechanical ventilation should promptly raise suspicion of an underlying esophagobronchial fistula.

## Conclusions

Esophagobronchial fistula is a rare but devastating complication occurring in the setting of thoracoabdominal aneurysmal disease and previous endovascular aortic repair. Progressive dysphagia may reflect aneurysmal esophageal compression and precede the recognition of an underlying esophagobronchial fistula. In patients who subsequently develop persistent bronchial secretions, recurrent pulmonary infections, or repeated unsuccessful weaning from mechanical ventilation, prompt multimodal evaluation with computed tomography, bronchoscopy, and upper gastrointestinal endoscopy is essential for timely diagnosis. Early recognition and multidisciplinary management are essential, although prognosis remains poor, particularly when advanced aortic disease and central airway involvement preclude definitive repair.
